# Microwave-assisted one-step synthesis of water-soluble manganese-carbon nanodot clusters

**DOI:** 10.1038/s42004-023-00983-6

**Published:** 2023-08-23

**Authors:** Nina Gomez-Blanco, Maurizio Prato

**Affiliations:** 1 Carbon Bionanotechnology Group, Center for Cooperative Research in Biomaterials (CIC biomaGUNE), Basque Research and Technology Alliance (BRTA), 20014 San Sebastián, Spain; 2 Department of Chemical and Pharmaceutical Sciences, INSTM – University of Trieste, Via L. Giorgieri 1, 34127 Trieste, Italy; 3 Ikerbasque, Basque Foundation for Science, 48013 Bilbao, Spain

**Keywords:** Synthesis and processing, Nanoparticles, Biomaterials

## Abstract

Using metal coordination to assemble carbon nanodots (CND) into clusters can enhance their photophysical properties for applications in sensing and biomedicine. Water-soluble clusters of CNDs are prepared by one-step microwave synthesis starting from ethylenediaminetetraacetic acid, ethylenediamine and MnCl_2_·4H_2_O as precursors. Transmission electron microscopy and powder X-Ray diffraction techniques indicate that the resulting clusters form spherical particles of 150 nm constituted by amorphous CNDs joined together with Mn ions in a laminar crystalline structure. The nanomaterial assemblies show remarkable fluorescence quantum yields (0.17–0.20) and magnetic resonance imaging capability (r_1_ = 2.3-3.8 mM^–1^.s^–1^). In addition, they can be stabilized in aqueous solutions by phosphate ligands, providing a promising dual imaging platform for use in biological systems.

## Abstract (web summary)

Using metal coordination to assemble carbon nanodots (CND) into clusters can enhance their photophysical properties for applications in sensing and biomedicine. Here, carboxylate groups on the surface of carbon nanodots serve as ligands for the coordination of manganese ions, enabling the assembly of optically and magnetically active CND clusters in a one-step microwave-assisted synthesis in water.

## Introduction

Carbon nanodots (CNDs), promising nanomaterials with size below 10 nm, have attracted considerable interest for their physico-chemical properties. Compared to traditional organic dyes and semiconductor quantum dots, CNDs are easy to prepare, highly soluble in aqueous media, photochemically stable and biocompatible^1–4^. In recent years, significant efforts have been devoted to the preparation of CNDs with metals to enhance their photophysical properties for use in sensing and biomedicine^5^. Introducing metals into the CNDs nanostructure may induce changes in the electron density distribution and energy gaps of CNDs^6^. For example, doping CNDs with metals can enhance quantum yield, produce multicolor emission, or even afford photocatalytic activity^7^. Moreover, the incorporation of magnetic metallic ions such as Gd(III) and Mn(II) into CNDs enables their use as contrast agents for magnetic resonance imaging (MRI)^8–12^. Conjugation of CNDs with magnetic nanoparticles is another strategy usually adopted to afford metal-CND hybrid platforms useful for multimodal imaging (optical/MRI)^13^.

Beyond their direct incorporation into functional materials, metals can also be exploited to design supramolecular assemblies composed by CNDs as nano-sized building blocks. In fact, the rich coordination chemistry of metals can drive the aggregation of CNDs in clusters of large dimensions and unique morphologies, provided that the right functional groups are available on the surface of the carbon nanomaterials. Metal-promoted formation of CND clusters is raising significant interest for application in medicine^14^. The increased size of CND composites potentially improves circulation time in the bloodstream and facilitates accumulation in tumors (EPR effect)^15^, in contrast to what typically occurs for smaller nanoparticles. Furthermore, disassembly and payload release in higher-order clusters can be tuned by external stimuli, such as pH.

These concepts were brilliantly demonstrated by Song, Cui and co-workers^16^ who employed Gd(III) ions to glue together CNDs and a photosensitizer (chlorine e6) in a self-assembled aggregate. The platform obtained through a multistep synthesis was capable of acting simultaneously as dual imaging (MRI and fluorescence) and photodynamic therapy agent in vivo. Nevertheless, procedures to favor self-organization of CNDs in aqueous solutions are still rare and most commonly achieved by the use of surfactants^17–19^. On the contrary, this type of processes is known to take place in organic solvents in the absence of metals when linkers such as PEG polymers are employed^20,21^.

In this context, we wondered whether or not we could capitalize on the expertise of our group in CNDs^22–24^ to devise novel and simple strategies for the preparation of Mn-CND clusters (**Mn**-**CND**-**Cs**) with potential application as dual fluorescence and MR imaging probes. To this aim, we envisioned that chemical groups such as carboxylates available on the surface of CNDs could serve as polydentate ligands for the coordination of metal ions, assisting the spontaneous one-pot and one-step assembly of large spherical CNDs architectures under optimized reaction conditions.

Herein, we demonstrate that such an approach is viable and that optically and magnetically active water-soluble **Mn**-**CND**-**Cs** can be straightforwardly obtained in situ using microwaves (MW). Our choice to focus on Mn-CNDs is motivated by the attractive properties of paramagnetic Mn(II) ions as MRI positive contrast agents, for liver imaging especially^25^. Indeed, Mn(II) offers a promising alternative to Gd(III) whose use in MRI is increasingly associated to neurotoxicity issues^26^. In this work, **Mn**-**CND**-**Cs** were successfully prepared in water, characterized, and then stabilized in phosphate buffer solutions.

## Results and discussion

**Fig. 1:**
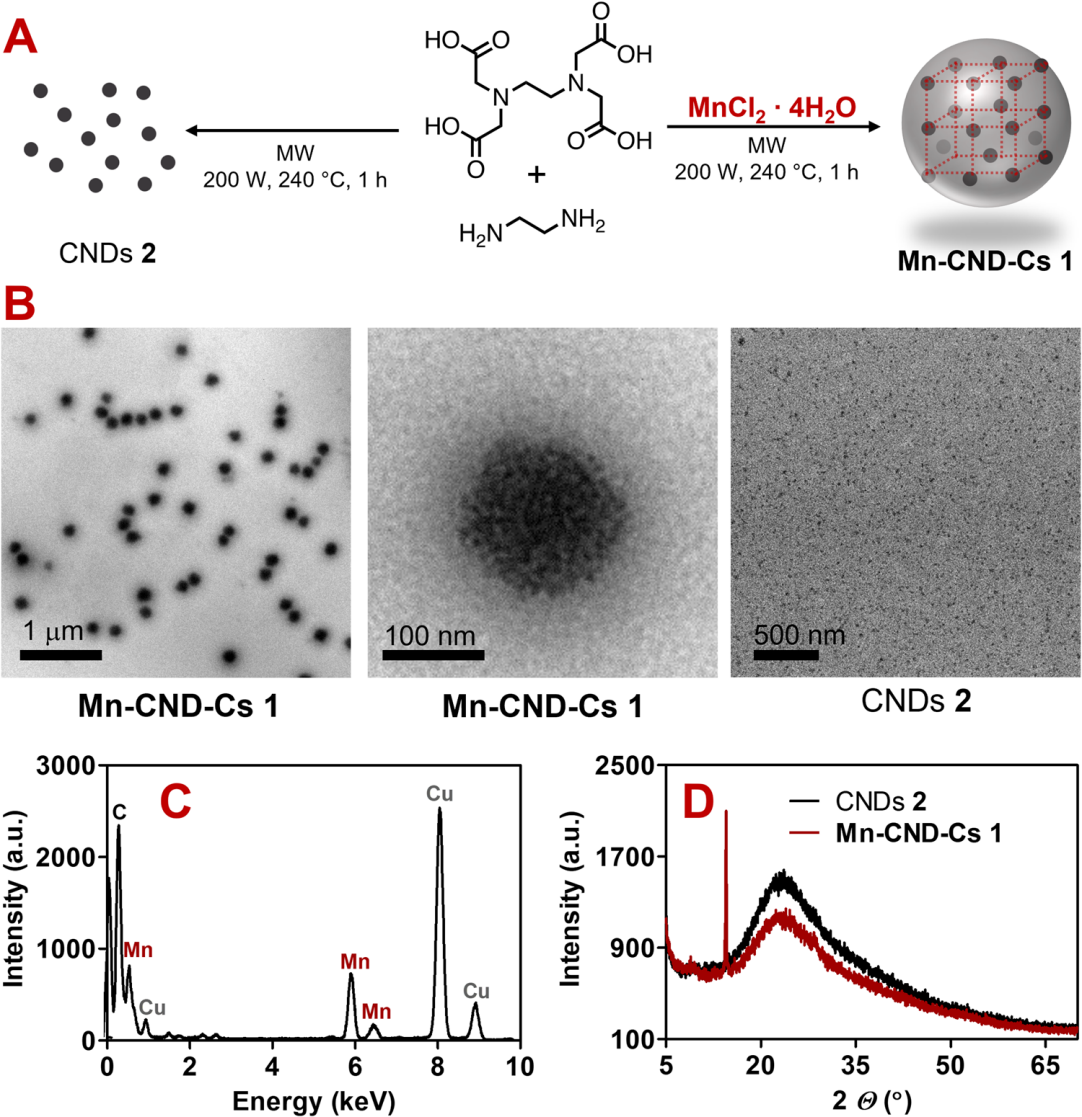
Preparation and characterization of materials. **A** Synthesis scheme, **B** TEM, **C** EDX, and **D** PXRD profile of freshly prepared samples of **Mn**-**CND**-**Cs 1** and CNDs **2**.

The synthesis of **Mn**-**CND**-**Cs 1** was achieved in one-step using a MW reactor under controlled conditions (240 °C, 27 bar, 200 W and 1 h heating time). We employed ethylenediaminetetraacetic acid (EDTA), ethylenediamine (EDA) and MnCl_2_·4H_2_O as precursors. Their molar ratio was carefully optimized to afford homogeneous nanometric particles after filtration and dialysis (Fig. 1A, B, see Supporting Information for details). For comparative purposes, we also carried out thermal carbonization reactions of EDTA and EDA in the absence of the Mn salt under the same experimental conditions (Supporting Information). Such control experiments led to the formation of amorphous CNDs **2** of 5–10 nm diameter, which displayed physicochemical features (vide infra) similar to others of the same kind previously reported^22^.

The initial characterization of **Mn**-**CND**-**Cs 1** was performed by Transmission Electron Microscopy (TEM), Energy-dispersive X-ray spectroscopy (EDX) and Powder X-Ray Diffraction (PXRD). Accordingly, TEM showed that the particles are uniform and adopt a spherical morphology with a narrow size distribution of 150 nm (Figs. 1B and S1). Notably, visual analysis of magnified TEM images and the presence of a broad band at high 2theta values in the PXRD, clearly demonstrate that the material incorporates amorphous CNDs similar to the ones (**2**) obtained in the control reactions without MnCl_2_ (Fig. 1D). EDX and STEM-EDX data confirm that the nanoclusters are constituted by Mn (Figs. 1C and S2). The presence of a narrow peak, positioned at 14.5° in the PXRD spectrum, indicates that **Mn**-**CND**-**Cs 1** has a laminar crystalline structure with an interlayer spacing of 6 Å (Fig. 1D). In accordance with literature examples, this diffraction pattern could be consistent with a crystalline framework corresponding to a MnO_6_ octahedral geometry^27–29^. ICP-MS established that the clusters contain 300 ng of Mn per mg of particles. The obtained assemblies display high water solubility and are not prone to further aggregation or precipitation.

The nature of the functional groups present on the surface of **Mn**-**CND**-**Cs 1** was investigated using X-ray photoelectron spectroscopy (XPS) and FT-IR (Figs. S3–S5). From the full-scan XPS spectrum of the material, Mn, O, N and C were detected with peaks at 643 eV (Mn 2p), 531 eV (O 1 s), 399 eV (N 1 s), and 285 eV (C 1 s), respectively. C, N and O configurations were analyzed by deconvoluting the corresponding signals. We determined three components for the C 1 s peak, consistent with sp^2^(C = O) at 289.5 eV, sp^3^(C–O, and C–N) at 287 eV and sp^3^(C–C) at 284.5 eV. The N 1 s spectrum could be deconvoluted into two peaks centered at 400.1 and 401.3 eV corresponding to NH_2_ and C–N–C groups, whereas the O 1 s spectrum well fit two components, which could be assigned to C–O and C = O groups. FT-IR further supports XPS data, showing that **Mn**-**CND**-**Cs 1** has several oxygenated groups on the surface, including carbonyls (broad peak centered at 1500 cm^–1^). Moreover, the colorimetric Kaiser test confirmed that approximately 1100 µmoles of primary amino groups per gram of material are present on the surface of these supramolecular structures.

As reported in Fig. S6, thermogravimetric analysis (TGA) of the obtained clusters determined a 70% weight loss between 100 and 470 °C, likely due to the loss of water molecules associated to the surface of the material, as well as of oxygen-containing groups. Total degradation of the sample was observed above 470 °C. An analogous TGA profile was recorded for CNDs, which however exhibit higher thermal stability as demonstrated by shift of the curve at higher temperatures (~140 °C).

All these results suggest that our synthetic strategy generates CNDs whose surface groups act as coordination partners for Mn ions, prompting the interconnection of CNDs with each other in a laminar crystalline structure, in accordance with PXRD (and TEM).

We can reasonably propose that EDTA provides carboxylic groups on the surface of the CNDs capable of chelating Mn ions, whereas EDA functions as nitrogen source that allows simultaneous nitrogen doping and surface passivation during the reaction, ultimately enhancing the fluorescence of **Mn**-**CND**-**Cs 1** (vide infra). Our group previously reported similar effects on the photophysics of other CND-based materials obtained from reaction mixtures containing EDA^22^. Consistently, control synthesis performed in the absence of EDA afforded an admixture of clusters and CNDs which, however, displayed poor emission features.

**Fig. 2:**
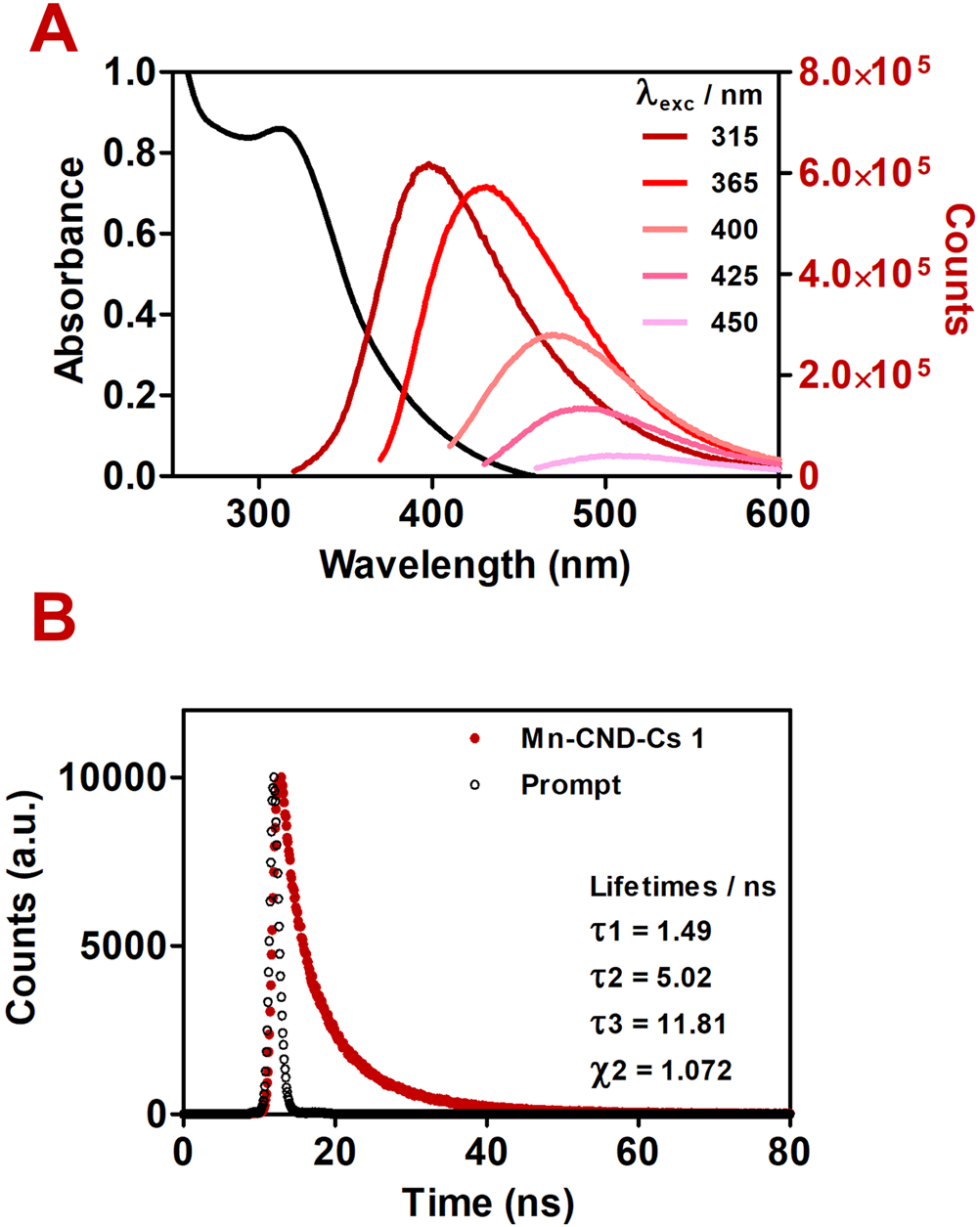
Photophysical properties of Mn-CND clusters (Mn-CND-Cs) 1. **A** UV-Vis absorption spectrum (black) and fluorescence spectra (colored) of freshly prepared samples of **Mn**-**CND**-**Cs 1** in water (298 K) at different excitation wavelengths and **B** time-resolved fluorescence-decay curve of a freshly prepared sample of **Mn**-**CND**-**Cs 1** in water (*λ*_exc_ = 340 nm and *λ*_em_ = 413 nm).

The absorption spectrum of **Mn**-**CND**-**Cs 1** exhibits a maximum at 315 nm and a long tail up to 450 nm. Emission spectra show the characteristic excitation-dependent profile typically observed for CNDs, with peaks shifting from 395 to 490 nm when the excitation wavelength changed from 315 to 450 nm (Fig. 2A).

As previously described^22^, intensities of these emission bands progressively decrease as they red-shift. The fluorescence quantum yield for the Mn-CND clusters was measured to be 0.17–0.20 in water and phosphate buffer (PB) solution (Fig. S7) using quinine sulfate as reference. We performed lifetime measurements using an excitation wavelength of 340 nm to monitor the decay of the emission band at 413 nm. A short (1.5 ns), intermediate (5.0 ns) and long (11.8 ns) component composed the lifetime at such emission wavelength in water (Fig. 2B), likely indicating the presence of multiple emission centers^10,30^. Comparison with the CNDs **2** prepared in this work shows that the two samples are very similar in their photophysics, including fluorescence lifetimes (Fig. S8). Notable differences for CNDs **2** compared to composite **1** are the red shift (13 nm) for the emission triggered by excitation at 365 nm^16,31^, and the somewhat lower fluorescence quantum yield (0.14) obtained for the same band. These results evidence how Mn ions and the assembly of CNDs in a multiparticle structure did not undermine fluorescence features in **Mn**-**CND**-**Cs 1**. Although not commonly observed, Liu et al.^8^ have previously reported that metal-driven aggregation of CNDs can result in a slight emission blue shift. This might be the result of a lower carbonyl content and a reduction of sp^2^ domains in the carbon matrix^32^. Accordingly, the ^1^H NMR spectrum of **Mn**-**CND**-**Cs 1** displays a set of resonances that can be ascribed to aliphatic protons and that are absent in the case of CNDs **2** (Fig. S9).

The stability of nanomaterials is one of the most important prerequisites for biomedical applications. For this reason, we investigated the behavior of **Mn**-**CNDs**-**Cs 1** in water and PB buffer over time, combining information from different techniques (Figs. S10 and S11). **Mn**-**CND**-**Cs 1** were less stable in pure water. ICP-MS showed that the release of Mn ions was complete after three days or even immediate when the clusters were treated with diluted hydrochloric acid. TEM confirmed that CNDs leached in the solution as a result of this process. Nevertheless, only a small change in the fluorescence intensity could be observed since free **2** and clustered CNDs **1** have similar quantum yields. The stability of Mn-CND clusters was significantly more pronounced in PB. For instance, the amount of free Mn determined at pH 5.5 by ICP-MS was only 15% after three days. Consistently, TEM revealed that no free CNDs are present in the solution, however **Mn**-**CND**-**Cs 1** dissolved in PB increased in size, reaching approximately 1 μm at pH 5.5. (Fig. S11). Importantly, such a change in dimension did not affect the emission properties of the material. We speculate that the observed increase in size for the clusters was driven by the coordination chemistry of Mn ions. Indeed, previous work highlighted that phosphate ions could bridge multiple Mn centers in metal complexes and small clusters^33–35^. Furthermore, it was reported that phosphate and metal ions such as Fe(III) or Cu(II) could act as bridges to connect CNDs together inducing aggregation^36^. A similar behavior would in principle favor the merging of several particles in large size, as observed for our **Mn**-**CND**-**Cs 1** under the tested conditions. Interestingly, when adding DMF (30%) to a PB solution (pH 5.5) in an attempt to crystalize these nanoarchitectures, we observed that **Mn**-**CND**-**Cs 1** slowly converted to capsule-like aggregates with a shell of 50 nm thickness (Fig. S12). The true composition of this shell is presently unknown; however, we believe it might be formed by coordination polymers/crystals involving Mn-phosphate and Mn-DMF bonds.

**Fig. 3:**
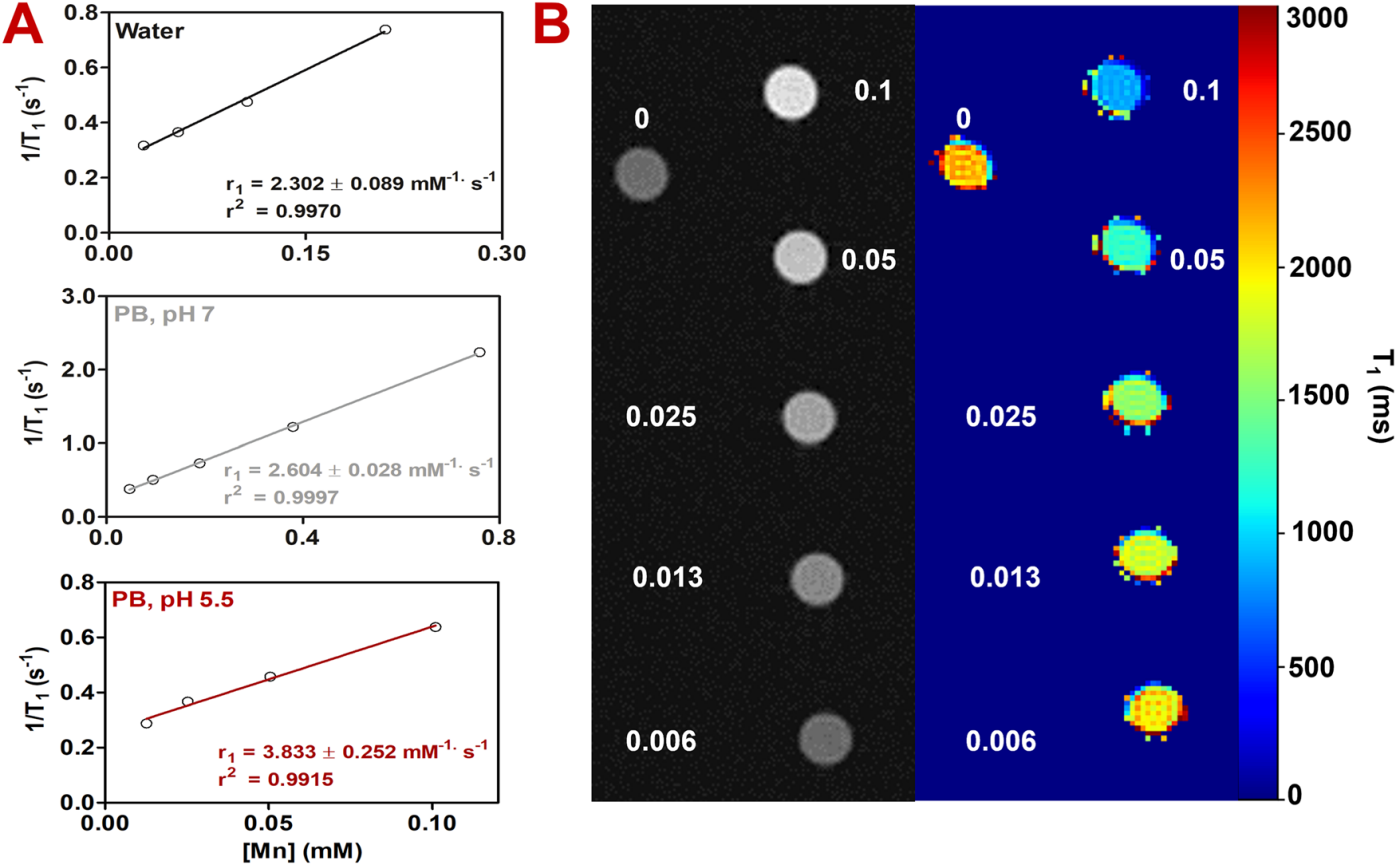
T1-weighted Magnetic Resonance studies. **A** Longitudinal MR relaxation curve recorded at 1.5 T of freshly prepared samples of **Mn**-**CND**-**Cs 1** in water and in phosphate buffer at pH 5.5 and 7; **B** T1-weighted MR and T1-map images recorded at 7 T of a freshly prepared sample of clusters (Mn concentration: mM) in PB buffer (pH 5.5) using a phantom.

The magnetic resonance (MR) imaging properties of **Mn**-**CND**-**Cs 1** were evaluated in terms of their relaxivity (*r*_1_) performance (Fig. 3). Measurements of the T_1_ relaxation times for freshly prepared samples at increasing Mn concentrations (up to 0.8 mM) were performed in pure water and in PB at pH 5.5 and 7 using a 1.5 T scanner. These experiments afforded *r*_1_ values of 2.302 ± 0.089 mM^–1^ s^–1^ in pure water, and 2.604 ± 0.028 mM^–1^ s^–1^ or 3.833 ± 0.252 mM^–1^ s^–1^ in PB at pH 7 and 5.5, respectively (Fig. 3A). The values obtained were in agreement with those reported for Teslascan©, a clinically approved Mn-based MRI contrast agent^37^, yet not as high as other Gd-based carbon materials, whose applicability however may be hampered by the increasing concerns on Gd toxicity^26^. We also examined the changes in relaxivity for the clusters in water and PB over time (7 days) to integrate the information obtained by TEM, ICP and fluorescence on the stability of these materials. **Mn**-**CND**-**Cs 1** preserved their ability to function as MR imaging agents (Fig. S13). The results show an increase of r_1_ values in water, consistently with the leaching of Mn(II) ions, whereas no variations in r_1_ were detected in PB over the time period monitored.

In addition, we investigated the capacity of the composites to function as contrast agents by acquiring T1-weighted MR images of a phantom at 7 T in PB (pH 5.5). As shown in Fig. 3B, good levels of contrast were observed for the material already at Mn concentrations of 0.1 mM.

## Conclusion

In summary, **Mn**-**CND**-**Cs 1** are a composite material obtained by microwave-assisted one-step synthesis using a straightforward procedure and readily available starting materials. Our methodology favors entrapping of fluorescent CNDs and Mn ions simultaneously with the assembly of the clusters. Carboxylic functional groups onto the surface of CNDs likely play an active role in the formation of higher-order structures.

The combination of paramagnetic Mn ions and blue-emitting CNDs makes **Mn**-**CND**-**Cs 1** a promising platform for use as dual fluorescence and MR imaging agents, as demonstrated by the characterization described in this contribution. The observed stability of these Mn-CND structures in PB is a suitable starting point to further develop this type of materials for application in biological systems. Future work will focus on investigating post-synthetic approaches based on ligand coordination chemistry with the aim of further improving the robustness of these materials or controlling their surface reactivity in biological media.

## Methods

### Materials

All chemicals were obtained from commercial sources. Ethylenediaminetetraacetic acid (EDTA, Sigma-Aldrich, ≥99.4%), ethylenediamine (EDA, Sigma-Aldrich, >99%) and manganese chloride tetrahydrate (Sigma-Aldrich, ≥99%) were used without further purifications. The Kaiser test kit and quinine sulfate were purchased from Sigma-Aldrich. Dialysis tubes with molecular weight cutoff of 0.5–1 KDa and filter membranes (0.2 µm, 17 mm, PTFE) were purchased from Spectrum labs and Thermo Scientific, respectively. Phosphate buffer (PB) solution at pH 5.5 and at pH 7 were purchased from Fisher Chemicals and Acros Organics, respectively. Ultrapure water obtained from a Millipore water purification system (Milli-Q, Millipore) was used in all experiments.

### Characterization

TEM studies were conducted on a JEOL JEM-2100F UHR electron microscope operating at 200 kV. Samples were prepared by depositing a drop (5 µL) of a solution of the sample onto a copper specimen grid coated with a carbon film (300 mesh) and allowing it to air dry.

EDX data were obtained using an EDX detector (Oxford, UltimMax). STEM images were performed from HAADF STEM detector. XPS experiments were performed using a SPECS Sage HR 100 spectrometer with a non-monochromatic X-ray source (Magnesium Kα line with an energy of 1253.6 eV and operating at 250 W), placed perpendicular to the analyzer axis and calibrated using the 3d^5/2^ line of Ag with a full width at half maximum (FWHM) of 1.1 eV. All measurements were made in an ultra-high vacuum (UHV) chamber at a pressure below 5 × 10^–8^ mbar. Gaussian–Lorentzian functions were used in the data fittings. The FWHM of all peaks were constrained while the peak positions and areas were set free. Samples were prepared depositing a drop of a solution containing the analyte on a gold support and allowing it to air dry.

X-ray powder diffraction patterns were collected employing a Philips X’pert PRO automatic diffractometer operating at 40 kV and 40 mA, in theta–theta configuration, secondary monochromator with Cu-Ka radiation (*λ* = 1.5418 Å) and a PIXcel solid state detector (active length in 2*θ* 3.347°). Data were collected from 5 to 80° 2*θ*, step size 0.026° and time per step of 1000 s at RT (total time 3 h). 1° fixed soller and divergence slit giving a constant volume of sample illumination were used.

TGA measurements were performed using a TGA Instrument Q500 (TA Instruments, Waters). Mn-CNDs clusters were dehydrated under the effect of heat and analyzed for TGA in nitrogen.

UV-VIS spectra were recorded at room temperature on a spectrophotometer Cary 5000 Varian. All the spectra were recorded at room temperature using 10 mm path-length glass cuvettes. The Kaiser test was carried out according to a published procedure^38^.

Fluorescence spectra were measured on a Fluorometer NIR (LS5-Edinburgh). Quantum yield measurements were performed using quinine sulfate in 0.10 M H_2_SO_4_ as the standard (Φ = 0.54, *λ*_exc_ = 360 nm) and following a procedure optimized by our group recently^24^. We employed excitation wavelengths of 315–450 nm, bandwidths of 1 nm for both excitation and emission and a 0.2 s dwell time. All the spectra were recorded at room temperature using narrow width 4 clear windows semi-micro glass cuvettes with 10 mm of optical path length.

FT-IR spectra were acquired in KBr using a Thermo Nicolet 5700 FT-IR spectrometer. The samples were analyzed for Mn using a iCAP-Q ICP-MS (Thermo Scientific, Bremen, Germany) equipped with an automatic sampler ASX-500 (CETAC Technologies, Omaha, USA). Quantification of Mn was carried out using the software Qtegra v2.6 (ThermoFisher, Bremen, Germany) monitoring isotopes ^55^Mn and as internal standard ^115^In.

^1^H NMR spectra were acquired on a Bruker 500 MHz Ultra Shield spectrometer. Spectra were calibrated using residual solvent signals.

Relaxation times (*T*_1_) were measured at 37 °C on a Bruker Minispec mq60 instrument operating at 1.47 T. The relaxivity values *r*_1_ were calculated through linear least-squares fitting of 1/relaxation time (s^–1^) versus the Mn concentration (mM). The MRI phantom experiments were performed on 7 Tesla Bruker Biospec 70/30 USR MRI system (Bruker Biospin GmbH, Ettlingen, Germany). T1 maps were obtained by using a spin echo sequence. Images were acquired at different TR values [7000.0, 3000.0, 1200.0, 900.0, 1800.0, 1200.0, 900.0, 700.0, 500.0, 300.0, 150.0, 55.0]. All data were acquired with TE effective (5 ms), RARE factor (1), FOV (38 × 38 mm), ACQ Matrix (160 × 160), RECO Matrix (160 × 160) and slice thickness of 1.50 mm. The total acquisition time was 94 min.

### Synthesis of Mn-CND-Cs and CNDs

All syntheses were performed using a CEM-Discover-SP microwave.

### Mn-CND-Cs 1

A stock solution of EDTA, EDA and MnCl_2_.4H_2_O (1:65:1 mol/mol) was prepared by dissolving 58.4 mg (0.2 mmol) EDTA, 869 µL (13 mmol) EDA and 39.6 mg (0.2 mmol) MnCl_2_.4H_2_O in 10 mL Milli-Q water. Then, 240 µL of such solution was heated in the microwave at 240 °C and 200 W for 1 h using the power cycling method (240 cycles total at 200 W, 15-s power interval, 5-s cooling interval, 240 °C as maximum temperature and 230 °C as minimum temperature). During the reaction, the solution color changed from brown to transparent and lastly to orange. Afterward, the reaction mixture was diluted with 5 mL of water and filtered through a 0.2 µm microporous membrane (PTFE). The filtrate was successively dialyzed against water through a dialysis membrane (cutoff = 0.5–1 KDa) for 2 days. The aqueous solution containing **Mn-CND-Cs 1** was finally lyophilized affording a dark orange oil (~15 mg). The amount of Mn was analyzed by ICP-MS.

### CNDs 2

A stock solution of EDTA and EDA (1:65 mol/mol) was prepared by dissolving 58.4 mg (0.2 mmol) EDTA and 869 µL (13 mmol) EDA in 10 mL MilliQ water. Then, 240 µL of such solution were heated in the microwave at 240 °C and 200 W for 1 h using the power cycling method (240 cycles total at 200 W, 15-s power interval, 5-s cooling interval, 240 °C as maximum temperature and 230 °C as minimum temperature). During the reaction, the solution color changed from transparent to yellow. Afterward, the reaction mixture was diluted with 5 mL of water and filtered through a 0.2 µm microporous membrane (PTFE). The filtrate was successively dialyzed against water through a dialysis membrane (cutoff = 0.5–1 KDa) for 2 days. The aqueous solution containing CNDs was finally lyophilized affording a light orange oil (~10 mg).

### Stability of Mn-CND-Cs 1 in solution

The degradation of Mn-CND clusters into single CNDs and Mn ions was monitored at room temperature in water and in phosphate buffer (PB) at pH 7 and 5.5 over time by TEM, fluorescence spectroscopy, ICP-MS and T1 relaxation time measurements.

The fractions of free CNDs and Mn ions for samples of **Mn-CND-Cs 1** in water or PB were separated using dialysis membrane (cutoff = 0.5–1 KDa). In order to quantify the percentage of Mn release over time, the separation was carried out for each time point. Solution aliquots retained in the dialysis membrane were first lyophilized and then their Mn content was determined by ICP-MS to evaluate the percentage of Mn released by **Mn-CND-Cs 1**.

### Supplementary information

Supplementary Information

## Data Availability

The authors declare that the data supporting the findings of this study are available within the article and Supplementary Information file or from the corresponding author upon reasonable request.
