# Supplementary material for: Microwave-assisted one-step synthesis of water-soluble manganese-carbon nanodot clusters
Source: Commun Chem. 2023 Aug 23;6:174. doi: 10.1038/s42004-023-00983-6 (PMC10447561; doi:10.1038/s42004-023-00983-6)
Supplement: Supplementary file 1 — Supplementary Information [file 42004_2023_983_MOESM1_ESM.pdf]

Supporting Information

Microwave-assisted one-step synthesis of water-soluble manganese-carbon nanodot clusters

Nina Gomez-Blanco,[a] Maurizio Prato*[a,b,c]

[a] *Carbon Bionanotechnology Group, Center for Cooperative Research in Biomaterials (CIC biomaGUNE), Basque Research and Technology Alliance (BRTA), San Sebastián 20014, Spain*

[b] *Department of Chemical and Pharmaceutical Sciences, INSTM – University of Trieste, Via L. Giorgieri 1, Trieste 34127, Italy*

[c] *Ikerbasque, Basque Foundation for Science, Bilbao 48013, Spain*

E-mail: prato@units.it

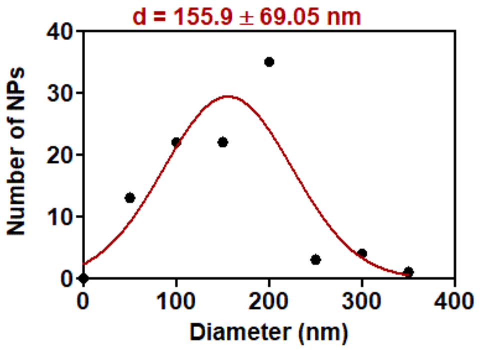

Figure S1. Average size distribution of a freshly prepared sample of **Mn-CND-Cs 1** in water as determined by TEM.

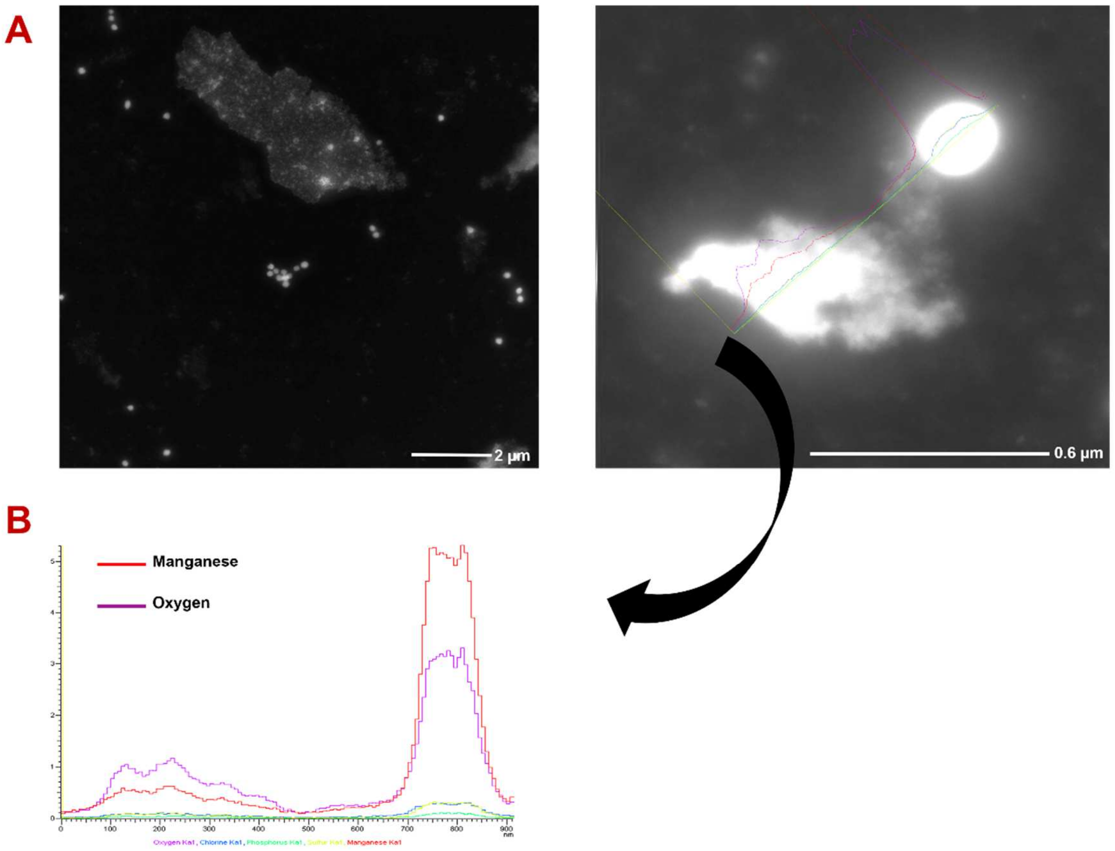

Figure S2. Representative HAADF-STEM images (A) and EDX line elemental map of corresponding O and Mn (B) of **Mn-CND-Cs 1** in water.

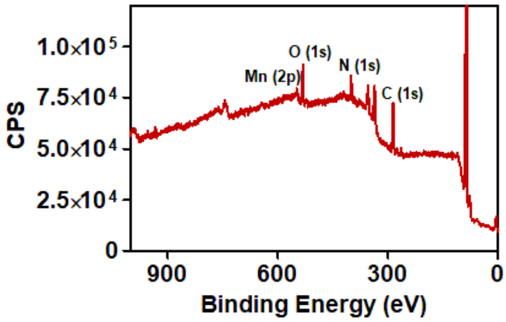

Figure S3. XPS survey of **Mn-CND-Cs 1**.

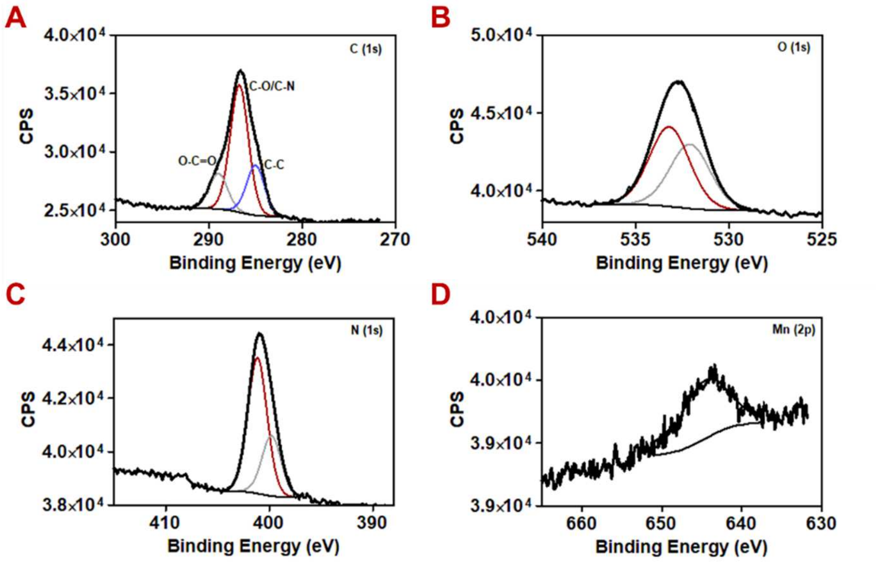

Figure S4. Deconvoluted XPS spectra of **Mn-CND-Cs 1**: (A) C 1s, (B) N 1s, (C) O 1s and (D) Mn 2p.

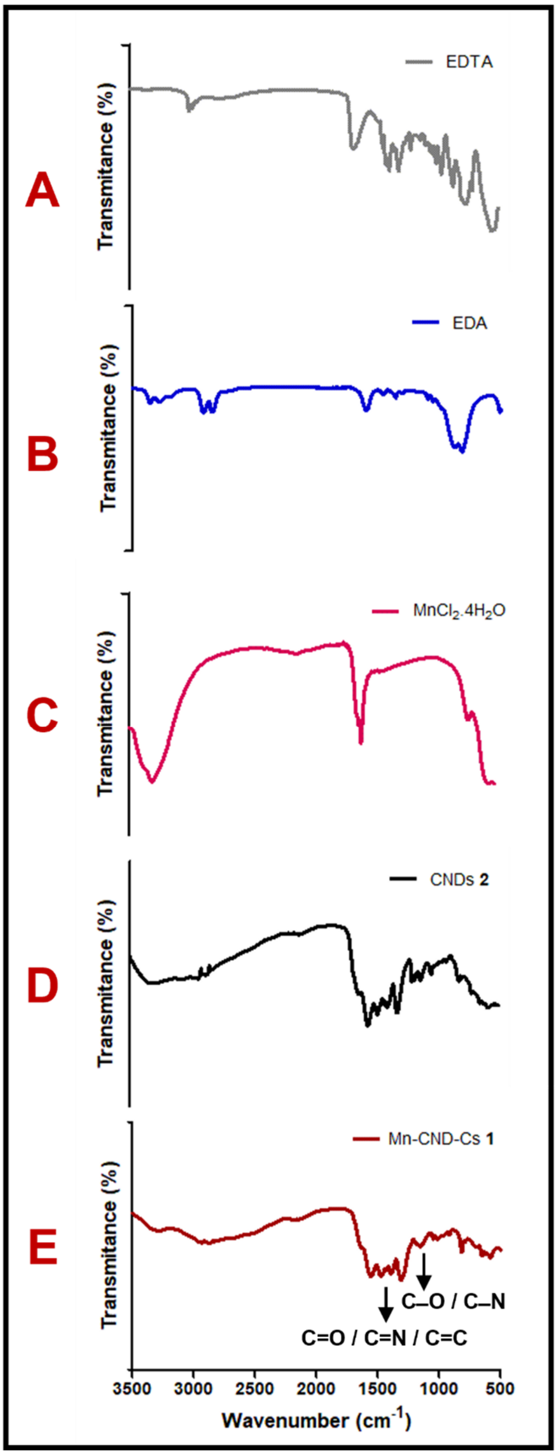

Figure S5. FTIR spectra of EDTA (A), EDA (B), $MnCl_2.4H_2O$ (C), CNDs **2** (D) and **Mn-CND-Cs 1** (E) in KBr.

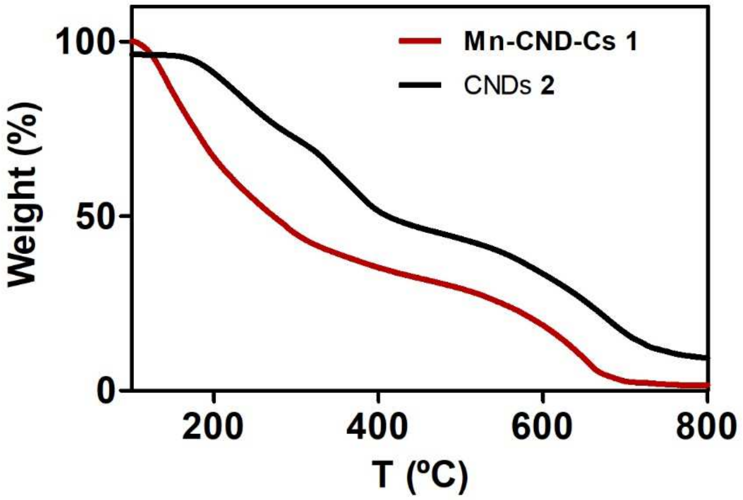

Figure S6. TGA curve of **Mn-CND-Cs 1** and CNDs **2** under nitrogen atmosphere.

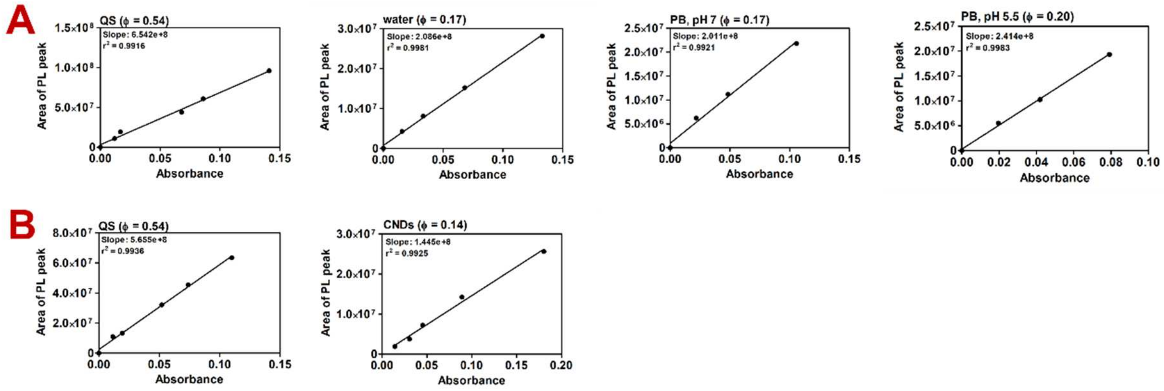

Figure S7. Quantum yield (Φ) obtained using quinine sulfate (QS) as the reference for A) **Mn-CND-Cs 1** in water and in PB at two different pH values (7 and 5.5) and B) CNDs **2**.

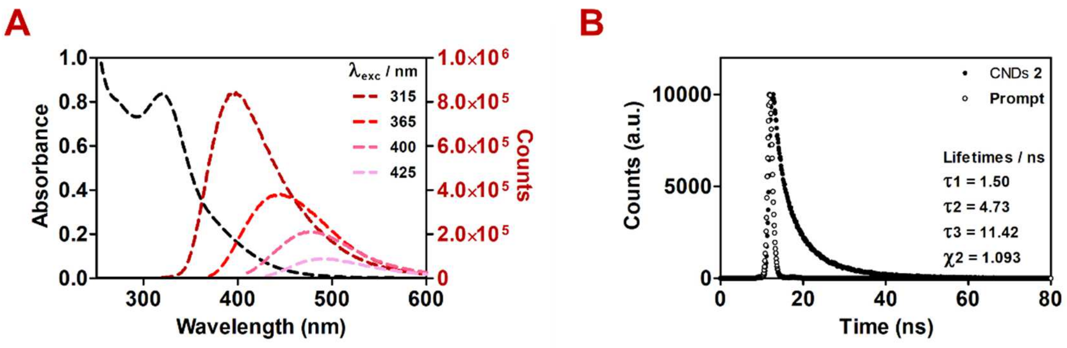

Figure S8. A) UV-Vis absorption spectrum (black) and fluorescence spectra (colored) of CNDs **2** in water (298 K) at different excitation wavelengths and B) time-resolved fluorescence-decay curve of CNDs (λ_{exc} = 340 nm) of the emission band at 409 nm.

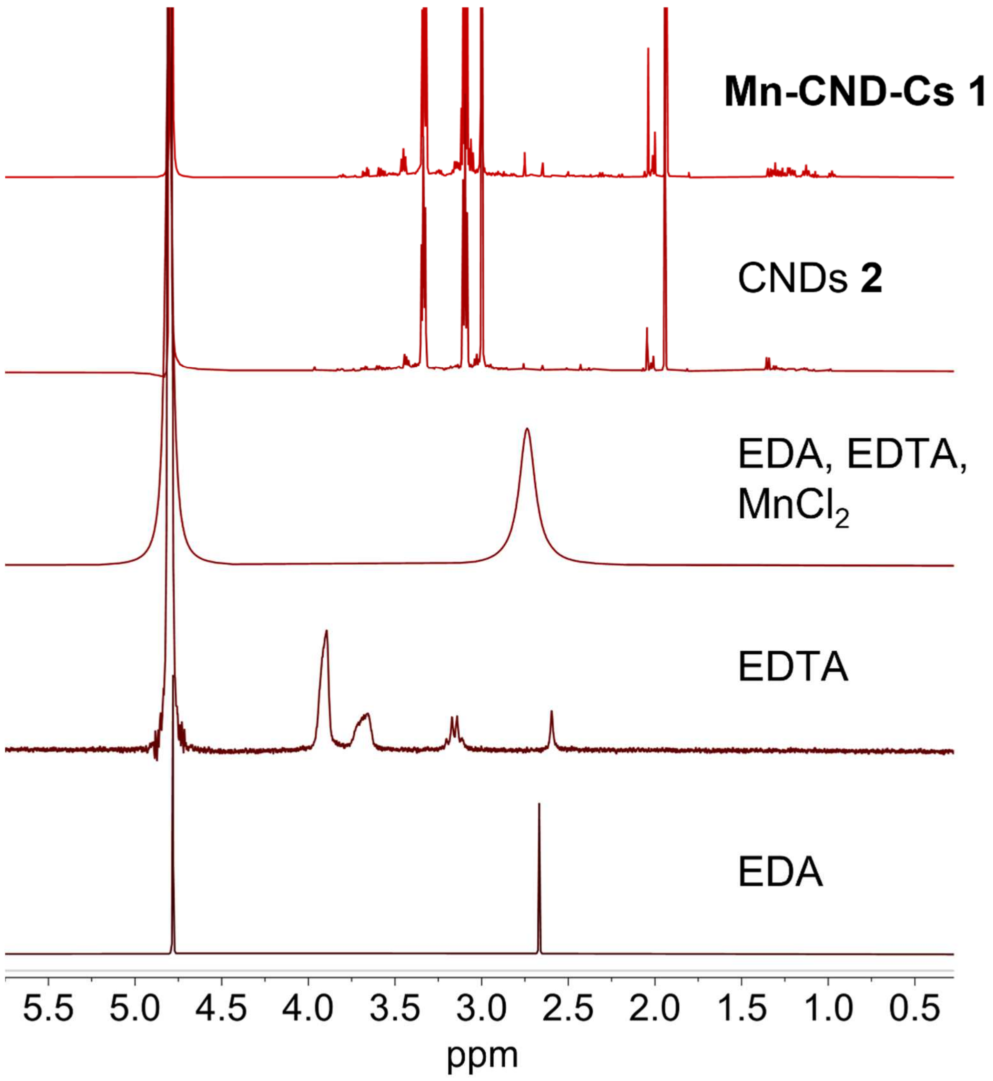

Figure S9. ^{1}H-NMR spectra of **Mn-CND-Cs 1**, CNDs **2,** and synthesis precursors in deuterium oxide.

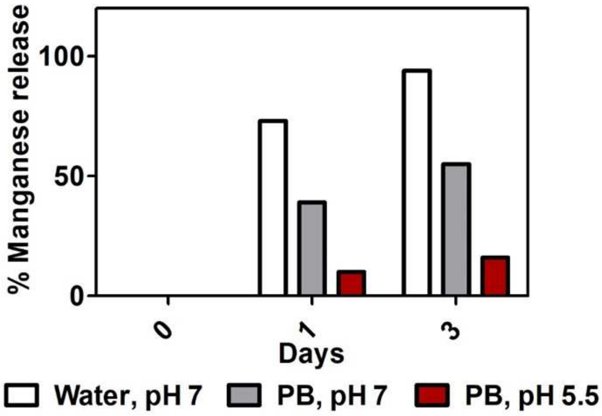

Figure S10. Time course of Mn release from **Mn-CND-Cs 1** in water and in PB at two different pH values, pH = 7 and 5.5.

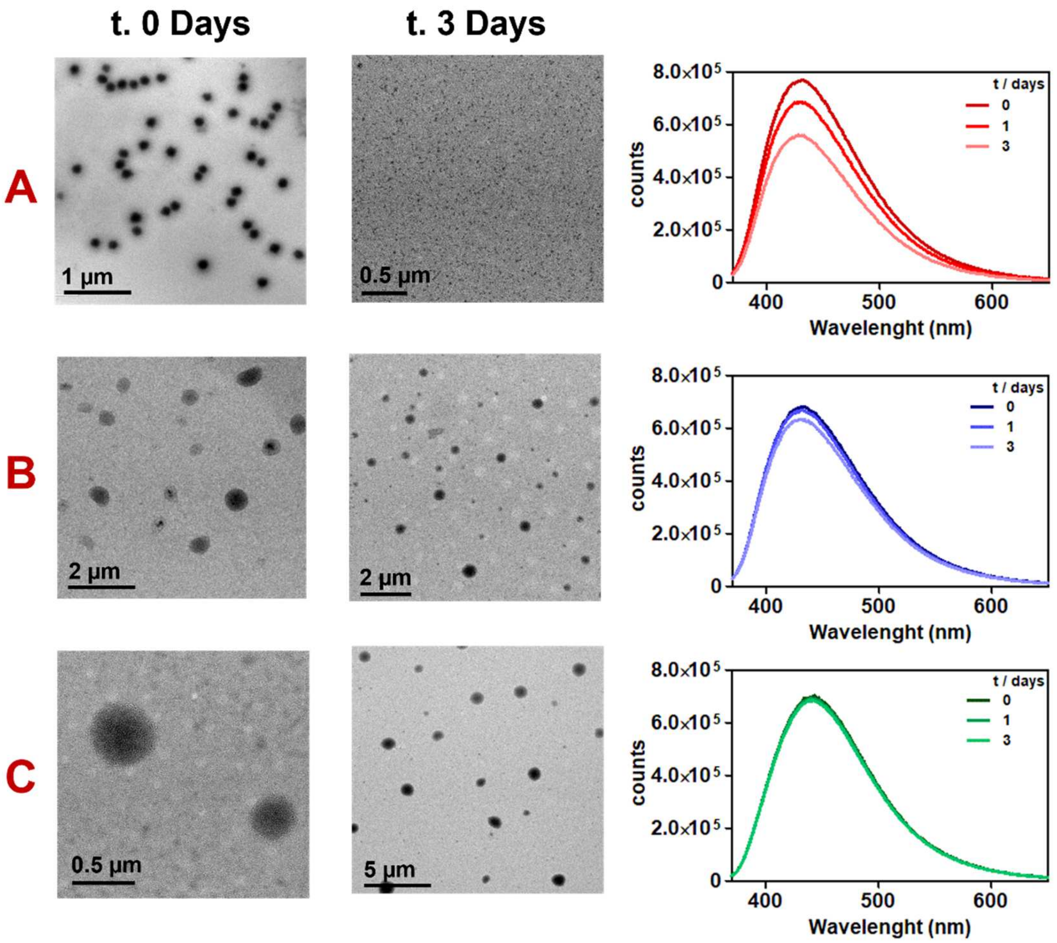

Figure S11. TEM images and fluorescence spectra (λ_{exc} = 365 nm) of **Mn-CND-Cs 1** recorded in water (A) and in PB at pH 7 (B) and pH 5.5 (C) over time.

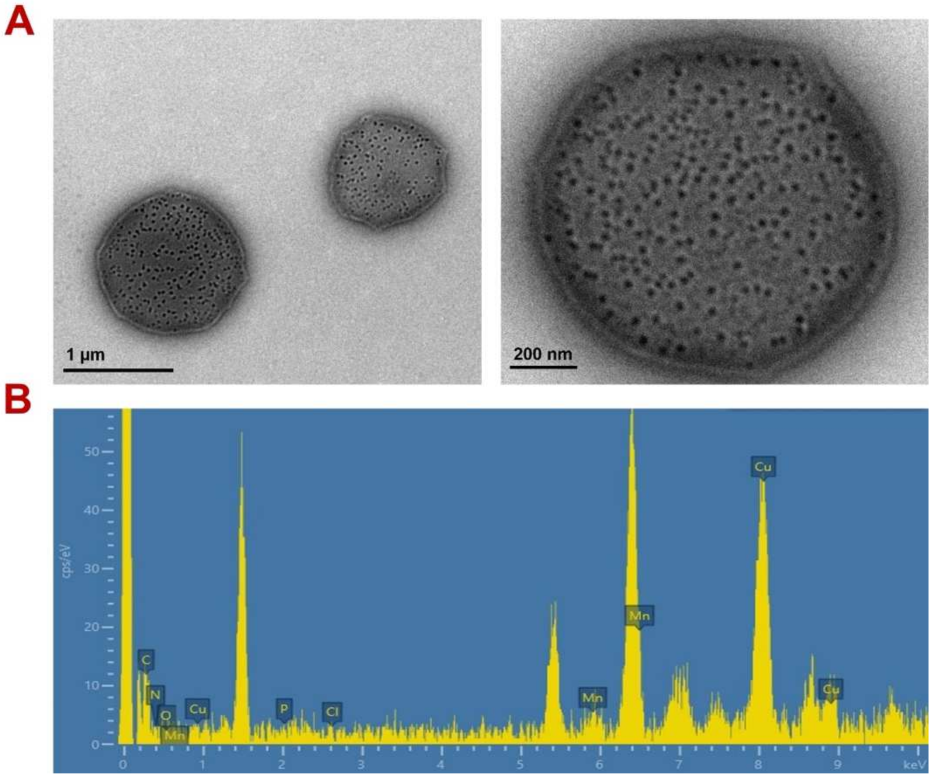

Figure S12. High-resolution TEM images (A) and EDX (B) of **Mn-CND-Cs 1** in PB at pH 5.5 (30% of DMF).

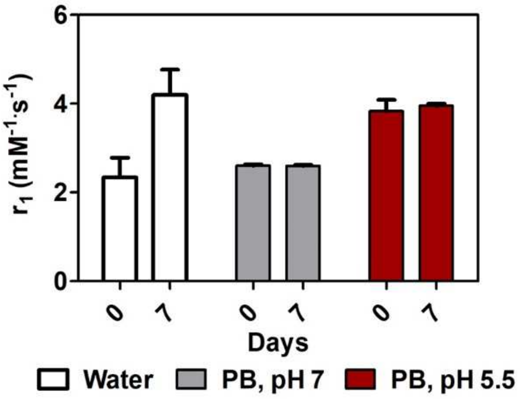

Figure S13. Longitudinal relaxivity value (r_1) obtained for a fresh stock of **Mn-CND-Cs 1** in water and in PB at two different pH values (pH = 7 and 5.5) and after 7 days in solution. Bar graphs with errors bars show the mean and standard deviation of at least 3 values of r_1 obtained for different fresh samples of **Mn-CND-Cs 1** in water and in PB at two different pH values (pH = 7 and 5.5) and after 7 days in solution.
